# The Right to Change Your Doctor

**Published:** 1915-11-20

**Authors:** 


### THE RIGHT TO CHANGE YOUR DOCTOR.

The right of the patient to change his doctor when it shall please him so to do has never been questioned by the medical profession, though in the mind of at least a section of the public this proposition may be disputed. The misconception arises from ignorance of the motive which underlies the professional demand that when such change is desired during the progress of an illness it shall be made openly and with the full knowledge of all the persons concerned. This motive is found in the welfare of the patient. It is in the patient's interest that the new adviser shall have exact medical information of the course of the illness, for without this he cannot be properly equipped for his responsibilities, and, to take but a single point, he may readily repeat methods of treatment which have already failed of success. Hence the condition that if a change is desired it must be made in circumstances which admit of consultation between the two practitioners concerned. The experience, it must be admitted, is a trying one, and needs excellencies of both tact and temper; but no practitioner with a sense of his own dignity can wish to continue professional relations when these are no longer accompanied by the confidence of the patient. Such, in brief, is the medical position in regard to the right of the public to a free choice of medical adviser.

The war, in addition to its numerous other effects, is likely to modify this principle, or, rather, to limit the application of it, and this in regard both to the classes who are absolutely free in their choice and to those who come under the National Insurance scheme. The latter, indeed, in spite of loud-sounding proclamations in the early days, have never enjoyed a full liberty. Not only was selection limited to doctors who entered their names on the panel, but when the selection had once been made change was only possible after due notice, and the opportunity occurred only once a year and at a fixed date. There was little consolation to a patient who believed himself to be seriously ill, and who desired another adviser, in the reflection that at the end of the year his desire could be gratified. We are not here criticising the arrangement but merely recording it. Now the difficulties of change of doctor are to be increased, and it will be necessary to show cause for the proposal. The aim is to conserve the interests of panel doctors who have undertaken military duties, and have necessarily left the care of their insurance patients in the hands of a substitute. These gentlemen are making great sacrifices for the nation's cause, and all will agree that, so far as possible, their interests should be protected even at the expense of the free will of the patient. There can be no such discipline for the more fortunate classes, but the moral claim to regard the position of medical practitioners who are serving with His Majesty's Forces will surely not be urged in vain. The doctors who remain at home are naturally anxious to act in all loyalty towards their absent colleagues, but the co-operation of the public is equally essential to success. The right to choose a medical adviser remains, but the occasion demands that this shall be exercised in such a manner as to show appreciation of the action of those who, in the stress of a national emergency, have risked their means of livelihood for the sake of the success which is vital to the liberties of us all.

